# Barriers and enablers to participation in physical activity among women diagnosed with ovarian cancer

**DOI:** 10.1007/s11764-023-01366-5

**Published:** 2023-05-12

**Authors:** Kellie Toohey, Catherine Paterson, Celeste E. Coltman

**Affiliations:** 1 Faculty of Health, University of Canberra, Bruce, Australian Capital Territory, Canberra, Australia; 2 Prehabilitation, Activity, Cancer, Exercise and Survivorship (PACES) Research Group, University of Canberra, Bruce, Australian Capital Territory, Canberra, Australia; 3 School of Nursing, Midwifery & Paramedic Practice, Robert Gordon University, Garthdee, Aberdeen, UK; 4 Canberra Health Services & ACT Health, SYNERGY Nursing & Midwifery Research Centre, Canberra Hospital, Garran, Australian Capital Territory, Canberra, Australia; 5 University of Canberra Research Institute for Sport and Exercise, University of Canberra, Bruce, Australian Capital Territory, Canberra, Australia

**Keywords:** Cancer, Exercise, Physical activity, Oncology, Therapy, Patient voice

## Abstract

**Purpose:**

Ovarian cancer is the leading cause of death among gynecological cancers, with low survival rates and a high disease burden. Despite the known benefits, most women reduce their participation in physical activity following diagnosis. Little is known about ovarian cancer survivors’ experiences of physical activity. The primary aim of this study was to explore the barriers and enablers to participation in physical activity among women diagnosed with ovarian cancer.

**Methods:**

A qualitative descriptive study design was conducted via semi-structured interviews with nine women diagnosed with ovarian cancer (stages I–IV; 40–77 years). The interviews took place at the participant’s home via telephone or online video conferencing software Coviu©. An inductive thematic approach was used. The organization and coding of data were completed using NVivo computer software (Version 12.6.0, QSR International Pty Ltd.). Weekly discussions occurred among the research team to ensure that themes accurately represented participant views. The consolidated criteria for reporting qualitative studies (COREQ) 32-item checklist were followed.

**Results:**

The main barriers to physical activity participation that emerged were (i) the lack of referral to an exercise professional within the multidisciplinary cancer team, (ii) fear of injury after surgery and during treatment, and (iii) treatment-related side effects. However, many of the participants perceived benefits of physical activity related to (i) enhanced physical and psychological health, (ii) improved cancer outcomes, and (iii) social benefits as key enablers of physical activity participation.

**Conclusions:**

Physical activity interventions for women with ovarian cancer should address the modifiable barriers identified in this study. A key focus should be to streamline timely referral pathways within the multidisciplinary team, including exercise professionals, dietitians, psychologists, and specialists nurses following a diagnosis of ovarian cancer. Further research and service development are needed to optimize supported self-management through (i) education about the importance of physical activity to both healthcare professionals and women alike, (ii) enhanced symptom management for women, which was identified as a barrier to participation, and (iii) the development of shared care plans and patient center goals to address any fears or concerns.

**Implications for cancer survivors:**

People diagnosed with ovarian cancer have low participation levels of physical activity. Cancer care professionals’ support could increase physical activity uptake and reduce some of the burden of an ovarian cancer diagnosis.

**Supplementary Information:**

The online version contains supplementary material available at 10.1007/s11764-023-01366-5.

## Introduction

Ovarian cancer is currently the seventh most common cancer worldwide [1] and the eighteenth most common cancer in women [2, 3]. Ovarian cancer is the fifth highest cause of cancer death among females [4], with poor survival outcomes [4, 5]. Almost 75% of women present with an advanced disease (Stage III or IV) [6], meaning that most women have a poor prognosis and a high risk of recurrent disease [2]. There are no screening tests for ovarian cancer, and the presentation of non-specific symptoms such as bloating, pelvic pressure, or pain and fatigue [6] have been attributed to delays in early diagnosis. Irrespective of the disease stage, women with ovarian cancer face invasive treatment regimes, which typically involve at least one major cytoreductive (debulking) surgery acompanied by multiple cycles of chemotherapy [7]. In the context of metastatic disease, women may undergo subsequent surgeries, further chemotherapy, immunotherapy or participate in clinical trials as a last treatment option [8]. Many women experience a high treatment burden [9], associated with high levels of distress, poor quality of life and reduced physical function during treatment, into survivorship and end-of-life care [10, 11].

Regular physical activity is recommended to mitigate cancer treatment side effects and improve physical and psychological well-being [12]. For women with ovarian cancer, clinical guidelines recommend that individualized exercise prescription is delivered under direct supervision of a qualified exercise professional if low physical function and high symptomology are present [12, 13]. In cancer care, physical activity is recommended as an adjunct cancer therapy, with emerging evidence supporting its efficacy in improving physical function and enhancing psychosocial health in women with ovarian cancer [14, 15]. Despite the reported benefits of regular participation in physical activity in other cancer groups [12], recent evidence has found that most women with ovarian cancer are insufficiently physically active following diagnosis [15–17]. Current guidelines recommend all people diagnosed with cancer participate in 150 min of moderate-intensity aerobic activity and two resistance sessions per week [12]. Recent research has shown that physical activity during and following treatment is safe, feasible, and effective in improving health outcomes in women affected by ovarian cancer [15, 18]. Disease progression, aggressive treatments, and high rates of recurrence have all been identified to negatively impact physical activity participation in women with ovarian cancer [14, 15]. Therefore, there is a clinical need to understand further why many women with ovarian cancer are physically inactive [15, 17]. Furthermore, understanding enablers of physical activity participation in this patient group are equally as important to develop delivery models of physical activity that are in keeping with the needs and preferences of women living with ovarian cancer.

Currently, there is little evidence on the reported barriers and enablers to physical activity among women with ovarian cancer [19], making it difficult to inform cancer service design and deliver appropriately tailored programs. Only two studies (*n* = 95 and *n* = 10) have been undertaken, which explored physical activity barriers perceived by women with ovarian cancer [16, 18]. These studies concluded that the barriers experienced were associated with disease or treatment-related side effects (such as pain, fatigue, and nausea) and personal attributes (such as lack of self-discipline, exercise not being a priority, and lack of interest) [16, 18]. Studies have explored women’s experiences of physical activity in mixed cancer participant groups [20, 21] and with all types of female gynecological cancers [22, 23]. However, these studies (participant number range 23–239) did not complete a sub-group analysis for individual cancer types [20–23], meaning that the interpretation of findings for women with ovarian cancer is problematic. Additionally, existing studies have used survey-based instruments for data collection, which limits clinical insights into women’s qualitative experiences of participating in physical activity [22, 23].

Given the challenges of a diagnosis of ovarian cancer and the limited knowledge of physical activity behaviors among this population cohort, further research is required to understand the unique needs and experiences of women diagnosed with ovarian cancer related to their engagement in physical activity. Such knowledge can be used to inform evidence-based interventions targeted at increasing physical activity participation among women diagnosed. Therefore, the current study aimed to explore barriers and enablers to participation in physical activity in women diagnosed with ovarian cancer.

## Methods

### Design

A qualitative descriptive design was conducted. Ethics approval was obtained from the University’s Human Research Ethics Committee (Project ID: 4507). Written informed consent was received from all participants prior to each interview [24, 25], and verbal consent was confirmed at the beginning of each interview prior to commencing the recording. The participants could withdraw from the study at any time without stating a reason. The consolidated criteria for reporting qualitative studies (COREQ) 32-item checklist were followed (see Supplementary Table 1) [26].

### Participants

A convenience sampling method [25] was used to recruit nine participants with a diagnosis of ovarian cancer (stages I–IV). Women were recruited via advertisements, flyers, word of mouth, social media posts, and invitation from oncology healthcare professionals and clinical care staff from a metropolitan regional cancer center in South East Australia. Women were eligible to participate in the study if they (a) had a primary or secondary diagnosis of ovarian cancer, (b) were aged 18 years or over, (c) had self-assessed proficiency in the English language, and (d) had access to a telephone or computer. The sample size was reflective of the small number of women living with an ovarian cancer diagnosis within the South East Australia region at the time of data collection.

### Data collection

Semi-structured interviews (mean time, 32 min) were conducted by telephone (*n* = 6) or online video conferencing (*n* = 3) in a one-on-one format from August 2020–September 2020. These methods of interviewing were chosen due to the suspension of face-to-face research due to the global coronavirus pandemic [27]. The interviewer was a female, qualified exercise physiologist (with Exercise and Sports Science Australia—ESSA) with experience in cancer care. The interviewer conducted several preparatory interviews and received mentoring and feedback from other members of the research team who had previous training and experience in conducting qualitative research. A semi-structured format was chosen to enable guided conversation around key issues informed by an interview topic guide (see Table 1). Discussions were fluid, and participants were encouraged to share their experiences beyond the established questions and probes. The interview topic guide was developed using the findings from a systematic review of the topic area [19].

**Table 1:** Semi-structured interview guide

Questions	Probes
Q1. What motivates you to be physically active?	Probe for: Quality of life Self-motivation Management of fatigue
Q2. Can you describe the barriers to physical activity that you have experienced?	Probe for: Disease-specific barriers versus environmental, social, and personal barriers Differentiate between current barriers and barriers pre-coronavirus
Q3. Are there any health concerns you think will be exacerbated or made worse by engaging in physical activity?	Probe for: Pelvic floor dysfunction, e.g., prolapse, incontinence Ongoing abdominal pain or discomfort post-surgery
Q4. What are your views on remaining physically active during cancer treatment?	Probe for: Beliefs around activity causing harm Education from members of the clinical care team
Q5. How confident do you feel about engaging in physical activity?	Probe for: Knowledge of appropriate activity Guidance from health professionals Independence and self-management strategies

The interviews took place at the participants’ home via telephone and online video conferencing software Coviu^©^. Prior to each interview, clinical, demographic, and physical activity data were obtained to describe the characteristics of the participants (see Table 2). The Active Australia Survey [28] was used to collect the physical activity levels of participants [29]. No other persons were present during the interviews. The researcher did not have a previous relationship with any of the participants, and the participants were not provided with any information about the researcher except for qualifications and primary contact details. The interviews were audio recorded using a digital recording device (Sony ICD PX-470). A reflective research diary was kept by the primary interviewer as a computer file on the university’s secure online database to capture initial impressions, thoughts, and early interpretations of the data.

**Table 2:** Participant demographic, medical, and physical activity characteristics (*n* = 9)

	*Mean* ± *SD (range)*
Age (years) at the time of the interview	65 ± 12 (44–77)
Total treatment duration (weeks)	29 ± 14 (18–56)
Physical activity levels	
Total weekly activity time (min)	236 ± 173 (35–480)
Total weekly activity sessions (number)	7 ± 4 (0–13)
Total vigorous* activity time (min)	27 ± 57 (0–180)
Total moderate** activity time (min)	6 ± 10 (0–30)
	*Number (%)*
Marital status	7 (78)
Married	1 (11)
Divorced	1 (11)
Widowed	
Highest educational attainment	
Diploma/advanced diploma	4 (44)
Bachelor’s degree	2 (22)
Post-graduate degree	3 (34)
Employment status	
Employed	3 (33)
Unemployed	1 (11)
Retired	5 (56)
Average yearly income ($, AUD)	
Not specified	4 (44)
0–24,999	1 (11)
25,000–49,999	2 (22)
50,000–74,999	1 (11)
75,000–99,999	1 (11)
Cancer stage at diagnosis	
Unknown	1 (11)
Stage I	1 (11)
Stage III	6 (67)
Stage IV	1 (11)
Treatment type	
Chemotherapy only	1 (11)
Chemotherapy + surgery	6 (67)
Chemotherapy + surgery + clinical trial	2 (22)

^*^Vigorous intensity physical activity: makes a person breathe harder or puff and pant; includes activities such as jogging, cycling, aerobics, and competitive sports (Australian Institute of Health & Welfare 2003)

^**^Moderate intensity physical activity: increases heart rate but does not necessarily make a person puff or pant; includes activities such as walking, golf, gentle swimming, and social tennis (Australian Institute of Health & Welfare 2003)

### Data analysis

The interviews were transcribed into written format immediately following each interview [30, 31]. The qualitative analysis adopted an inductive thematic approach, as described by Braun and Clarke [30] (see Table 3 for details). The organization and coding of data were completed by hand and using NVivo computer software (Version 12.6.0, QSR International Pty Ltd.). Weekly discussions occurred with the research team to ensure the established themes accurately represented participant views. The transcripts were not returned to the participants for comment as the research team had audio recordings and notes to check accuracy. No repeat interviews were carried out.

**Table 3:** Phases of thematic analysis (adapted from Braun and Clarke [30])

Phase	Description
Familiarization of data	Familiarization of data was completed while transcribing the interviews into written form, which involved reading and rereading the data. The interviewer kept a research diary to note down initial ideas. The research team also familiarized themselves with the transcripts by reading and rereading them
Generation of initial codes	Two of the research team identified features of the data relevant to the primary research question and noted down initial codes. Any discrepancies in codes were identified and discussed among the research team to meet a consensus
Identifying themes	Two of the research team reviewed codes and began to organize data into preliminary themes according to similarities. At this stage, all data was split according to barriers and enablers. The research team discussed the preliminary themes to ensure a group consensus was reached. A thematic map was developed by collapsing codes into preliminary themes
Reviewing themes	The research team further refined themes by ensuring the coded data extracts were accurately categorized into the appropriate theme. Coded extracts under each theme were reread, and the thematic map was refined to ensure it accurately represented the entire data set
Defining and naming themes	A short description for each theme was developed
Writing report	Relevant extracts linked to the research question and literature were identified, and a full report was written by the interviewer. Guidance was received by the broader research team

## Results

### Participants

Nine women (44–77 years) diagnosed with ovarian cancer consented to participate in the study. Most participants (*n* = 6) had completed both chemotherapy and radiation treatment and were stage III (*n* = 6). There was large variability between age and treatment duration for the participant sample (Table 2).

### Themes

**Fig. 1:**
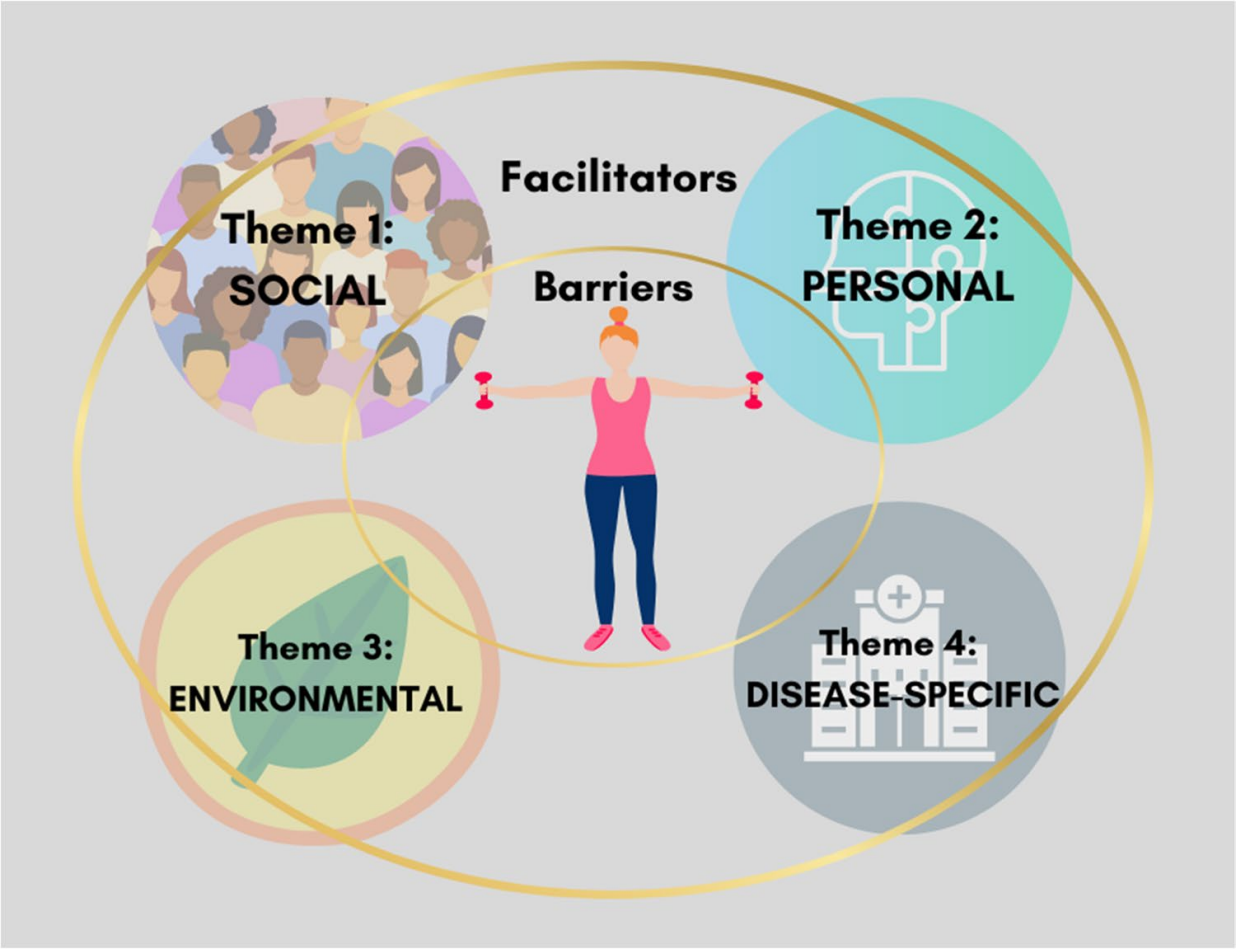
Emergent themes for the barriers and enablers to physical activity participation

Four overarching themes were identified for both barriers and enablers to participation in physical activity (Fig. 1). Each theme is described in the subsequent sections, with direct quotes from the interviews used to illustrate participant experiences.

### Barriers to physical activity

#### Theme 1: social

All participants reported that social factors negatively impacted their engagement in physical activity. Participants specifically highlighted a lack of physical activity advice from their clinical care team members and the absence of referrals to an exercise professional (such as an accredited exercise physiologist or physiotherapist) with experience in cancer care. The absence of this referral pathway meant that many participants had not completed targeted rehabilitation following major abdominal surgery and, subsequently, often remained inactive throughout chemotherapy (typically an 18-week period). In addition, participants reported a lack of coordination from the clinical care teams, particularly following initial surgery.

*“The problem, as you probably already know from speaking to other women, is that you have to go to Sydney for your surgery. So, once you leave there, you’re basically on your own. You don’t have that contact with the team, I guess. Like I wasn’t offered… a cancer psychologist or anything like that. Which my girlfriend who had breast cancer had all this support for her, through her surgery and everything that was here. Whereas I didn’t have physio or anything, once I left hospital that was it. So, I self-referred, got my GP (General Practitioner) to refer me to an EP (Exercise Physiologist), but um, it certainly wasn’t suggested by any of the doctors or the oncologist or anything.”*

Family and household responsibilities were another consistent topic that emerged as a social barrier to physical activity. Several participants with school-aged children spoke about their sense of responsibility to contribute to their households, which took priority over participation in physical activity and structured exercise. One participant also reported that being a single parent meant there was very little time to engage in regular physical activity.

*“I guess I just wanted to help again around the house. So rather than focus on exercise it was more just when I had the energy, I wanted to feel like I was back to contributing to the household, you know, whether it was cooking a meal or putting a load of washing on.”*

One participant also mentioned the beliefs of family and friends discouraged her from engaging in physical activity, even when she was feeling motivated and well. She expressed her frustrations regarding their recommendations to rest and suggested further education should also be provided to family and friends about the importance of regular physical activity throughout cancer treatment.

*“It’s a big factor with people around you who think you rest and you should not do this and not do that. And I think that was quite a barrier, that they need educating that you know what’s good for yourself and that exercising when you can and when you’re motivated is good, and not to put you off. And not tell you that you should go and lay down and you shouldn’t go out in the garden or you shouldn’t do this, you should rest.”*

#### Theme 2: personal

All participants highlighted personal factors that were perceived as barriers to physical activity participation. Personal factors included both physical and psychological. One-third of participants spoke about physical activity not being a priority, particularly during weeks when chemotherapy side effects peaked, which limited participant’s ability even to complete daily tasks and errands.

“*You have a priority list of things you need to get done and you can quite easily push the exercise to the side*.”

Almost all participants mentioned fear and uncertainty about resuming physical activity following surgery. Participants were concerned that rushing back into physical activity following surgery may increase pain or cause damage to the incisional sites. This lack of certainty around appropriate activity following surgery meant participants lacked self-management support because they often felt poorly informed to engage in any physical activity except for walking.

“*Obviously, straight after the surgery, you’re worried that you’re going to pop everything open if you’re too physical.”*

“*You just have this kind of mental fear of, you don’t know, is there any damage to any internal stitching or other bits and pieces.*”

#### Theme 3: environmental

Several environmental factors were highlighted by participants as barriers to regular physical activity engagement. These included location, access to facilities, time, weather, cost, as well as the global coronavirus pandemic. Several participants lived in regional areas and reported difficulty in accessing facilities. Only two participants mentioned time as a barrier to physical activity, both of whom had additional household responsibilities as single parents and carers for elderly family members. For some participants, hot weather prevented them from engaging in physical activity, while others reported cold weather as a deterrent (i.e., they were more likely to go for a walk when the weather was sunny and warm).

*“I think this weather, the weather will play a big part. Ah you know. ‘cause now it’s coming into the nice warmer weather … where you know, like in the middle of winter, you don’t want to get up in the morning and put your walking shoes on and go outside.”*

Cost was only highlighted as a barrier by one participant who was unable to attend a formal exercise facility for this reason. The unusual circumstances surrounding the global coronavirus pandemic were mentioned by many participants; however, it was only perceived as a barrier to structured exercise and group classes rather than general physical activity.

“*The covid itself was a barrier to doing … organized exercise. But we could still walk.*”

#### Theme 4: disease specific

Disease-specific factors were reported by all participants as barriers to physical activity and were categorized under surgery, chemotherapy, and clinical trials. Surgery was perceived as a major physical activity barrier, with many participants reporting incisional discomfort, poor recovery, pelvic floor dysfunction, and persistent pain. One participant also spoke of incisional discomfort, which prevented her from wearing an appropriate underwire sports bra, limiting her to low-intensity activity.

*“I found, the pain lasted for a long time, to the point where, even when I was out walking – I tried to walk most days as soon as I could – I’d have to hold my abdomen. It just sort of felt like things were rolling around the inside and banging around the outside, and every time they did that, it was painful.”*

The transition back to physical activity following surgery was also mentioned by the participants as an area of need for increased support and clinical intervention.

*“I think it would be good if we were steered in a direction towards like an exercise plan or something, then at least we could have something to get our teeth into that’s sort of new and specific to the problem, and after the surgery perhaps, that might just give you something to get your teeth into and maybe work from there.”*

Chemotherapy also resulted in a range of side effects that discouraged participants from participating in physical activity. Side effects such as anemia, blisters, peripheral neuropathy, fatigue, gastro-intestinal issues, neutropenia, pain, and weakness were all common barriers among participants. The presence of chemotherapy-induced peripheral neuropathy also resulted in impaired balance for some participants, which elicited fears of falling and being in a gym around other people.

“*One of my issues is that I’ve got, you know, peripheral neuropathy in my feet. And my balance isn’t good, and I tend to trip.*”

Participants were also concerned about their compromised immune system, with several participants experiencing neutropenia throughout their chemotherapy. There was concern that physical activity would decrease immune function further and, therefore, increase the likelihood of contracting a virus.

“*Then because of white blood cells and the immune system, I felt I couldn’t go out because I’d get a cold. So, it really stopped me doing a lot of exercise.*”

Participation in clinical trials was common among participants, which also resulted in side effects such as severe anemia and fainting episodes, meaning participants felt unsafe engaging in physical activity, particularly outside of the home.

“*If we turn to the side effects of the clinical trial, I felt it was quite unsafe to be out and about.*”

### Enablers to physical activity

#### Theme 1: social

Social enablers were related to other individuals, organizations, and health care professionals. The primary factors classified under the social umbrella were support from family, friends, spouses, and partners; group exercise classes; knowledgeable health professionals; and access to or the provision of physical activity education. It was evident from the majority of interviews that support from family members (specifically their husbands or partners) was a key enabler to participating in regular physical activity. Many participants reported their husbands or spouses engaged in physical activity with them, which enhanced motivation and provided a sense of security, particularly when exercising outside of the home.

“*I can mainly think of things that weren’t barriers, like my husband has just been super supportive and encouraging.*”

Attending group classes or exercising with other individuals was also perceived as a key physical activity enabler. Participants reported the accountability and consistency of a group class or walking club supported ongoing physical activity adherence and enhanced motivation. Attending a class with individuals of a similar demographic and physical capability was also mentioned as an enabling factor.

“*I find it difficult just to rely on myself or motivate myself to go and exercise. I need that accountability, I suppose, of other people expecting me to be there.*”

Support from appropriately qualified and knowledgeable exercise professionals was also key to facilitating participation in regular physical activity. Participants reported this support enabled them to engage in group classes or an exercise program, knowing they were safe and that exercises could be modified if required due to side effects or preexisting conditions. Women were more inclined to remain physically active throughout chemotherapy and following surgery if they were well supported by qualified health professionals.

“*It’s a real enabler I think to know that you’re in safe, knowledgeable hands.*”

“*I feel well informed and supported, and I’m really pleased I got the exercise physiologist helping, cause otherwise, I wouldn’t have really known what I was doing.*”

Despite the lack of education reported by some participants, this was not the case for all interviewed. Some participants had received education from medical and healthcare professionals, including medical oncologists, physiotherapists, exercise physiologists, and clinical care nurses. However, the information provided was reported to be quite general and not specific to what exercise or physical activity they could do. The timing of this education was inconsistent among participants, with some receiving information during chemotherapy, while others spoke about the provision of education immediately following their diagnosis. Some participants also sought information through websites, pamphlets, and radio and television programs.

#### Theme 2: personal

Personal factors, which were perceived as enablers of physical activity, included physical and psychological. All participants spoke about the importance of regular physical activity for general health benefits and overall well-being. One-third of participants outlined the importance of physical activity for weight maintenance and weight loss. Over half of the participants spoke about physical activity being essential for mental health benefits and psychological well-being.

“*For me, personally, it’s essential for my mental health. I go crazy if I can’t get some physical activity every day.*”

Having a preexisting relationship with physical activity before cancer diagnosis meant some participants found it easier to remain active throughout treatment. Having already built the habit of regular physical activity meant these participants found it less difficult to get motivated when it was already part of their usual lifestyle.

“*I’ve always been physically active… it’s part of my lifestyle.*”

Some participants spoke about the importance of attitude in staying physically active. Participants who reported a positive attitude toward their diagnosis and overall life were more likely to be motivated to participate in physical activity throughout their cancer trajectory.

*“I think having a determined and a determinedly positive attitude, even with the knowledge that the stats are not great… will enable me to keep doing exercise. Because if you just sort of said well, it doesn’t matter, I’m gonna be dead in two years, you wouldn’t do it. Or you may not do it. So I think even though I say I’m not motivated to do a lot of exercise I think, a positive attitude generally, if one can do it, would probably be an enabler of exercise.”*

Not only was a positive attitude an enabler but also the opinion that physical activity would improve resilience and the ability to cope during times of reduced health or disease progression.

*“I work very hard at being positive in the times I’m feeling well, and I know that by doing exercise in those periods in particular, that it will see me be able to cope better with the times when I’m less able, put it that way.”*

#### Theme 3: environmental

Geographical location was reported to be the leading environmental enabler of physical activity engagement. All participants lived in South East Australia, where there is an abundance of outdoor spaces and nature reserves to suit varying levels of fitness and physical capabilities.

“*We’re very fortunate here that, you know, you can go walking either in the street or down by the lake or wherever you want.*”

Access to these locations during the coronavirus lockdown also enabled participants to remain active and physically distanced from others.

“*For a start, we live in a beautiful area; we can walk in the bush and get to the bush in a few minutes. So we had lots of socially isolated walks on Black Mountain.*”

#### Theme 4: disease specific

Disease-specific enabler was the final theme that emerged from the interview data. Many participants had a second or third recurrence of ovarian cancer and therefore spoke about the importance of physical activity to reduce the risk of future cancer recurrence.

*“I think it really, it gave me a real big wake-up call, having the recurrence. And I just thought nup I can’t just go on the way I was… thus now I’ve just now had a twenty-kilo weight loss journey. I just have to give myself the best chance I have, I can, of it not coming back a third time. So, and I don’t want to be laying there at the end going oh shit, why didn’t I try a bit harder. So yeah, I think that was probably why I did exercise more. I just realized I had to, absolutely had to.”*

Participants also spoke about the importance of physical activity to improve cancer outcomes, specifically to reduce the burden of side effects and maximize the effectiveness of treatment.

*“It’s well, mainly just about dealing with the cancer but yeah, I guess being aware that it’s both, it’s while I’m having the chemotherapy, minimizing the side effects and maximizing the effectiveness. So both of those things are motivating.”*

Additionally, physical activity was a motivating factor improving general well-being and quality of life despite a diagnosis of advanced ovarian cancer.

“*I’m improving … my ability to stay well while living with ovarian cancer.*”

## Discussion

To the best of our knowledge, this is one of the first qualitative studies which has identified barriers and enablers to physical activity participation among women diagnosed with ovarian cancer. The findings of this study provide the needed insight into the physical activity experiences of these women, identifying key areas for consideration when designing interventions and ways to increase participation. The implications of the unique findings of this study as they related to women diagnosed with ovarian cancer are discussed below and summarized in Table 4.

**Table 4:** A summary of the barriers and enablers to physical activity among women diagnosed with ovarian cancer as identified in this study. The barriers and enablers unique to women diagnosed with ovarian cancer compared to other cancer types are highlighted in bold

Theme	
	Barriers
Social	• Lack of physical activity advice from the clinical team • No referral to an exercise professional with exercise experience, such as an exercise physiologist • **No rehabilitation following abdominal surgery** • Lack of care coordination • **Family and household responsibilities** • **Being a single parent, prioritizing family over physical activity** • **Family beliefs, including recommendations to rest rather than move**
Personal	• Physical activity not being a priority, particularly during treatment cycles • **Fear and uncertaintly about resuming physical activity following sugery** • **Fear about physical activity increasing pain and causing damage** • Lack of self-management support
Environmental	• Location, access to facilities • Weather • Costs
Disease specific	• **Post surgery, incisional discomfort wearing specific clothing** • **Lack of support in the transition to return to physical activity** • Chemotherapy side effects’ negative impact on physical activity • The compromised immune system, concerns that exercise would reduce immune function • Side effects from clinical trials
	**Enablers**
Social	• **Support from family (specifically husband or partner)** • Group classes (particularly with the same demographic) • Access to physical actvity education • Support from qualified health professionals • **Modified exercise delivered by qualified professionals (particulary during treatment cycles**
Personal	• **Physical activity routine before diagnosis** • **A positive outlook toward diagnosis** • **A belief that physical activity improves resilience and the ability to cope**
Environmental	• Access to outdoor locations such as nature reserves
Disease specific	• **The knowledge that physical activity improves cancer outcomes** • **A positive experience of the benefits of physical activity including reduced burden and severity of treatment-related side effects** • Understanding that physical activity improves positive feelings of well-being

The lack of clear physical activity education and referrals to exercise professionals was consistently reported by all participants as a major barrier following surgery and during treatment, which is a finding similar to mixed cancer cohorts in women [20]. Participants commonly reported that physical activity advice was too generalized and not specific to their individual circumstances. Most women were encouraged to walk immediately following surgery (by either their surgeons or hospital physiotherapists); however, only one participant was referred to an exercise professional for specialized programming by their medical oncologist. This finding is concerning, given that existing literature has reported that support and approval from medical oncologists have been identified to be facilitators of physical activity participation [32], meaning that many people are likely not to participate without this support. Furthermore, participants felt support from a health professional was required, with two participants choosing to self-refer to an exercise physiologist following surgery. Accredited exercise physiologists and physiotherapists with experience in cancer are well placed to address the needs of women with ovarian cancer [13].

Consistent with previous research, a significant personal barrier to physical activity was fear and uncertainty following surgery [16]. Many women highlighted the need for increased support to transition back into physical activity after surgery and reiterated the need for medical and health care professionals to provide reassurance to them. The limited research in this area demonstrates the vital role that health professionals play in enabling physical activity participation [16, 18, 19].

Known barriers to physical activity include weather extremes and cost [16, 22], which were also identified in this study. Some participants additionally reported time as a barrier (particularly those with school-aged children) and challenges with juggling household responsibilities, including caring for elderly parents. Women reported that it was difficult to access an exercise facility with appropriate supervision; this was also identified by Mizrahi et al. [16]. To improve physical activity participation, interventions must be accessible. Tele-health platforms may provide an opportunity to increase accessibility and, therefore, participation in physical activity [33, 34].

Disease and treatment-specific factors are known barriers to participation in physical activity [16, 18, 20, 22], which were consistent with the results of this study. The qualitative design of the current study provided new insights into the type, location, and timing of reported side effects. Most women reported pain immediately after surgery, specifically at and around the incisional site, and pain with activity (e.g., walking and light core exercises). Women also identified concerns with persistent fatigue following surgery, during treatment, and post-treatment, decreasing their likelihood of physical activity participation.

All study participants reported that exercising with other individuals (such as family, friends, or other cancer survivors) was an enabler of physical activity. Tyrrell et al. (2014) reported the importance of social support, showing that 50% (*n* = 239) of gynecological cancer survivors valued peer support during a physical activity program [23]. Given that a lack of social support is a known barrier to physical activity participation [16], future programs might consider the importance of developing group-based physical activity interventions for women with ovarian cancer. Additionally, women value knowledgeable health professionals qualified to deliver physical activity programs [22, 23]. This study identified that not all participants had access to accredited exercise physiologists and physiotherapists, identifying a gap in existing clinical services.

Participants of the current study perceived physical activity intensity as both a barrier and enabler to physical activity participation. Most women with ovarian cancer tend to prefer low to moderate-intensity physical activity, with higher intensities perceived as a barrier by participants identified in other studies [23, 35]. Evidence supports the use of high-intensity interval training in female cancer survivors [36, 37], which may provide a time-efficient physical activity option [12, 38]. However, individualized pre-screening should take place before prescribing high-intensity interval training to ensure patient safety and preferences.

This research was conducted during the coronavirus pandemic [27]. The pandemic changed the delivery of programs and exercise interventions for individuals, including people affected by cancer [33, 39]. Most of the study participants reported that they remained somewhat active throughout lockdown periods.

## Limitations

The results of this study must be interpreted in light of the limitations. Firstly, the small sample size reflected the low number of women in the South East region of Australia, where the study was conducted, living with an ovarian cancer diagnosis at the time of data collection. Even though there were a small number of participants, women’s voices are needed to move the field of physical activity for people diagnosed with ovarian cancer forward. It is acknowledged that recruitment bias is possible because we were unable to capture reasons for non-participation due to ethical approval restrictions. Secondly, all the study participants were located in the South East region of Australia, limiting the transferability of findings to other geographical locations. Finally, the sample was biased in favor of women who had stage III ovarian cancer and were treated with both chemotherapy and radiation treatment, providing little insight into the experiences of women with other clinical characteristics. However, our study sample is representative of the majority of women diagnosed with ovarian cancer [5, 40].

## Future research directions

Future studies are recommended to investigate care coordination, education, and the development of flexible referral pathways that cater to the diverse group of women diagnosed with ovarian cancer and their individual needs. Finally, future research should investigate the perceived barriers and enablers to physical activity and provide timely, appropriate referrals to qualified professionals within multidisciplinary teams.

## Conclusion

When considering physical activity interventions for women with ovarian cancer, health care teams should address the barriers and enablers identified in this study. A key focus should be to streamline timely referral pathways within the multidisciplinary team of exercise professionals, dietitians, psychologists, and specialist nurses following a diagnosis of ovarian cancer. Further research and service development are needed to optimize supported self-management through (i) education about the importance of physical activity to both healthcare professionals and women alike, (ii) enhanced symptom management for women, which was identified as a barrier to participation, and (iii) the development of shared care plans and patient center goals to address any fears or concerns about physical activity reported by the women.

## Supplementary Information

Below is the link to the electronic supplementary material.

Supplementary file1 (DOCX 19 KB)

## Data Availability

Data will be available with reasonable request.
