# Supplementary material for: Barriers and enablers to participation in physical activity among women diagnosed with ovarian cancer
Source: J Cancer Surviv. 2023 May 12;18(4):1252–63. doi: 10.1007/s11764-023-01366-5 (PMC10175906; doi:10.1007/s11764-023-01366-5)
Supplement: Supplementary file 1 — Supplementary file1 (DOCX 19 KB) [file 11764_2023_1366_MOESM1_ESM.docx]

**Supplementary Table 1** Consolidated criteria for reporting qualitative studies (COREQ) checklist: 32-item checklist

| **No. item** | **Description** | **Reported on page** |
| --- | --- | --- |
| **Personal characteristics** |  |  |
| 1. Interview/facilitator | The author who conducted the interviews | Page 22 |
| 1. Credentials | The researcher’s credentials | Page 5 |
| 1. Occupation | The interview’s occupation at the time of the study | Page 6 |
| 1. Gender | Male, female or non-binary | Page 5 |
| 1. Experience and training | Experience and training of the researcher | Page 6 |
| **Relationship with participants** |  |  |
| 1. Relationship established | Relationship prior to study commencement | Page 6 |
| 1. Participant knowledge of the interviewer | Knowledge about the researcher | Page 6 |
| 1. Interviewer characteristics | Characteristics reported about the interviewer | Page 5 |
| **Theoretical framework** |  |  |
| 1. Methodological orientation and theory | The methodological orientation underpinning the study | Page 5–6 |
| **Participant selection** |  |  |
| 1. Sampling | Method of participant selection | Page 5 |
| 1. Method of approach | How participants were approached | Page 5 |
| 1. Sample size | Number of participants in the study | Page 5 |
| 1. Non-participation | Number of participants who refused to participate or dropped out | Page 5 |
| **Setting** |  |  |
| 1. Setting of data collection | Location of data collection | Page 5 |
| 1. Presence of non-participants | Presence of other individuals at the time of data collection | Page 5 |
| 1. Description of sample | Important characteristics of the sample | Page 7, Table 2 |
| **Data collection** |  |  |
| 1. Interview guide | Interview guide and prompts used | Page 6, Table 1 |
| 1. Repeat interviews | Statement of whether repeat interviews were conducted | Page 8 |
| 1. Audio/visual recording | Type of interview recording | Page 6 |
| 1. Field notes | Description of field notes made during or after the interview | Page 6 |
| 1. Duration | Duration of the interviews | Page 5 |
| 1. Data saturation | Discussion around data saturation | Page 5 |
| 1. Transcripts returned | Return of transcripts to participants | Page 8 |
| **Data analysis** |  |  |
| 1. Number of data coders | The number of data coders who coded the data | Page 8, Table 3 |
| 1. Description of the coding tree | Description of the coding tree | Page 8, Table 3 |
| 1. Derivation of themes | Identified in advance or derived from the data | Page 8, Table 3 |
| 1. Software | Software used to manage the data | Page 8 |
| 1. Participant checking | Feedback from participants | Page 8 |
| **Reporting** |  |  |
| 1. Quotations presented | Participant quotations presented to illustrate the themes | Page 10–17, Results |
| 1. Data and findings consistent | Consistency between data presented and the findings | Page 10–17, Results |
| 1. Clarity of major themes | Major themes clearly presented | Page 9, Fig. 1 |
| 1. Clarity of minor themes | Description of minor themes or categories | Page 9, Fig. 1 |
